# COVID-19 and coping: Absence of previous mental health issues as a potential risk factor for poor wellbeing in females

**DOI:** 10.1016/j.dialog.2023.100113

**Published:** 2023-02-08

**Authors:** Gemma L. Witcomb, Hannah J. White, Emma Haycraft, Clare E. Holley, Carolyn R. Plateau, Chris J. McLeod

**Affiliations:** School of Sport, Exercise and Health Sciences, Loughborough University, Loughborough, Leicestershire LE11 3TU, United Kingdom

**Keywords:** pandemic, wellbeing, coping, longitudinal

## Abstract

COVID-19 has caused unprecedented disruption to everyday life. Unsurprisingly, this has resulted in increased prevalence of poor mental wellbeing. While previous mental health issues have been consistently flagged as a risk factor, the absence of these may also leave individuals vulnerable due to a lack of psychological coping strategies. This study explored the change in symptoms of anxiety, depression, and trauma in 167 females who provided data at four timepoints over the course of the first year of the pandemic. There was a significant effect of time on the extent of the change in depression but, for all wellbeing measures, those with current or previous mental health issues experienced a similar magnitude of change as those with no previous issues. This suggests that low-risk individuals may be faring worse, relatively. Ensuring that this group is not overlooked will be imperative in protecting and re-building the wellbeing of the nation.

## 1 Introduction

In March 2020 the COVID-19 virus sweeping the world caused the UK to instigate its pandemic response and enforce its first national lockdown. Over the next 24 months, fluctuating levels of restrictions were imposed, both at local and national levels. The resulting disruptions have had significant negative consequences for the majority of the population in all core areas of life, including employment, education, and health and social care [1].

Undoubtedly, the effects of the pandemic will be felt for many years, with both economic recovery and the backlog of routine healthcare treatments presenting significant challenges. In addition, early estimates of the cost of treating conditions associated with long COVID (i.e. longer term health issues resulting from having the COVID-19 infection) put it at around 30% of the pandemic-related health burden [2]. However, this is likely to be a vastly conservative estimation, since this does not include mental health related conditions [3] which are likely to be significant. Indeed, a number of short longitudinal studies, conducted at the start of the pandemic, have shown increases in common mental health disorders and psychological stressors (e.g., [4]), highlighting that mental wellbeing is a key casualty in the pandemic.

In attempting to understand how the pandemic has affected wellbeing (and may continue to do so), it is important to consider risk factors. Indeed, the pandemic has perfectly highlighted the plethora of existing social inequalities that influence health and wellbeing [5], so much so that in the US understanding of external sources of inequality has improved and advocacy efforts have grown [6]. Gender is a significant factor in this [7,8] and the “COVID Motherhood Penalty” is a term that has been coined to describe the negative impact on working mothers’ employment and finances as a result of the disproportionate childcare burden experienced during the pandemic [9]. Indeed, a study from Ireland found that the pandemic forced a redefinition of family dynamics in many households, which resulted in women taking on additional roles (e.g., teacher) and a disproportionate amount of domestic labour, leading to poorer wellbeing and increased negative emotion [10]. A number of studies have reported gender differences in wellbeing outcomes, including women reporting greater caution and more compliance with restrictions than men [11], greater worry about their partner’s and children’s health [12], and increased feelings of disconnection and loneliness [13]. Given that health workers were working under conditions of unprecedented personal threat and the risk of PTSD tends to be greater in women (due to differing cognitive schemas [14,15] and the influence of sex hormones and menstrual cycle on emotional processing [16]), it is perhaps not surprising that female health workers and physicians are reported to have suffered greater negative effects on wellbeing compared to male [17].

Another important consideration in understanding the impact of the pandemic on wellbeing is whether an individual has any pre-existing vulnerability (i.e., a previous or current mental health issue). Indeed, a history of mental health disorders has been identified as a significant risk factor in the epidemiology of pandemic-related ill health [18] and a short three-wave study over the first 6 weeks of the pandemic in the UK (March 2020–May 2020) found that pre-existing mental health issues were a risk factor for poorer mental health outcomes [19], as was younger age, greater deprivation, and being female. Therefore, many existing social inequalities, highlighted by the pandemic [5], also add an intersecting risk [8]. However, focusing on the exacerbation of existing inequalities and risk factors may be missing an additional risk factor; lack of experience or skills to manage wellbeing.

Many psychological factors interact to influence how an individual reacts to a negative life event. The construct of ‘coping’ has been used to describe different patterns of behaviours and cognitive strategies that individuals may engage in – either consciously or unconsciously – as a way to escape from threat, be it external or internal [20]. Coping can be both adaptive (e.g., changing mental schemas to incorporate something positive from stressors) and maladaptive (e.g., restricting or controlling food intake and body shape in order to feel a sense of control in an uncertain situation). Maladaptive coping strategies are linked to poorer mental wellbeing outcomes and are a core focus of treatment for mental health concerns. For example, Cognitive Behavioural Therapy (CBT; [21]) is concerned with challenging negative (maladaptive) patterns of thought. Once these patterns of coping are recognised, challenged, and new patterns of thinking become internalised and ingrained, individuals are better able to cope with future negative events. Thus, receiving treatment for mental health problems can equip a person with the psychological skills and knowledge to draw upon during future negative life events. Ultimately, they may cope better than someone who has not required intervention from a mental health professional.

What underlies the development of particular coping strategies is unclear. Resilience is a key factor involved in wellbeing and is related to the ability to withstand, cope with, and bounce back from adverse life events, adapting positively to the negative event. A recent global survey conducted by Wong et al. [22] reported that lower resilience was associated with more maladaptive coping behaviours (e.g., increased alcohol intake, reduced exercise) and that risk factors for lower resilience were related to social inequalities, such as female gender, younger age, financial insecurity, and physical health. A UK cross-sectional study undertaken at the start of the first lockdown (May 2020) also found that psychological flexibility - the ability to respond flexibly to life demands in order to achieve personally meaningful life-goals - was related to positive wellbeing and lack of negative mental health [23]. Psychological flexibility is suggested to predict coping responses; those who are more inflexible may be more likely to engage in maladaptive coping strategies, such as avoidance [24]. Thus, flexibility in approach to challenges is likely to offer some protection against pandemic-related poor ill-health. However, a key question is whether receiving previous or current treatment for poor mental wellbeing (which may suggest inflexibility and poor coping) represents a vulnerability, as has largely been hypothesised - or whether experience of engagement in treatment affords some protection for maintaining wellbeing during the exceptional circumstances of a 21^st^ Century pandemic.

Based on this, we hypothesised that previous / current mental health issues may be less of a risk factor during the COVID-19 pandemic than was largely feared, due to the likely adaptive flexibility and coping skills that previous / current treatment would have provided. Thus, this study sought to explore, longitudinally, how mental wellbeing changed across the first 12 months of the pandemic for females, specifically comparing those who were currently or previously seeking treatment for a mental health issue to those who had never sought treatment. Of interest was whether, within the context of a pandemic, lack of current / previous mental health treatment may, ironically, be a greater risk factor for poorer wellbeing.

## 2 Method

This data was collected as part of a study exploring mental wellbeing, eating behaviour, physical activity, and sleep during the pandemic in the UK. Only the data related to our hypothesis of mental wellbeing is reported here.

### 2.1 Participants and timepoints

Participants were recruited via opportunity sampling on social media to take part in an online survey, initially at three timepoints (T1: March/April 2020 – first national lockdown; T2: June/July 2020 – eased restrictions; T3: October/November 2020 – tiered local restrictions and second national lockdown). As the pandemic continued, the data collection was extended to fourth timepoint (T4: February/March 2021 – third national lockdown). Participants who completed T1-T3 and T4 were entered into a prize draw, each time to win one of eight £50 Amazon vouchers. Participants provided informed consent and ethical approval for the study was obtained from the *(BLINDED)* University Ethics Approvals (Human Participants) Sub-Committee (2020-1378-181).

### 2.2 Measures

Each online survey included demographic questions and the following mental wellbeing measures which participants were instructed to answer thinking about their feelings over the past two weeks:

**Hospital Anxiety and Depression Scale** [25]. This 14-item questionnaire consists of 7 items measuring symptoms of anxiety and 7 items measuring symptoms of depression, each answered on a 4-point (0-3) response scale, giving an overall total of 0-21 per subscale. A score of 0-7 is considered to indicate the absence of a mood disorder, 8-10 as possible presence, and 11-21 as probable presence. It has been used widely in a range of settings and populations and has excellent validity (e.g., [26]).

**Screening Questionnaire for Disaster Mental Health** [27]. This is a 12-item measure with two sub-scales to assess symptoms of PTSD (9 questions) and symptoms of depression (6 questions; note that three questions are counted and scored in both the sub-scales). In this case, only the 9 items assessing PTSD were used and were amended to be specific to the pandemic (e.g., Do you get upset when something reminds you of the COVID-19/Coronavirus pandemic?”). Items have yes (1) or no (0), responses, with an overall score of 0-3 indicating slightly affected / little possibility of PTSD, 4-5 moderately affected, and 6-9 severely affected / possible PTSD. The tool has good reliability and has been validated in other languages (e.g., [28]).

**Treatment-seeking behaviour.** Two yes / no questions asked about treatment-seeking behaviour. Specifically, “Have you ever sought treatment for a mental health problem?” and “Are you currently seeking treatment for a mental health problem?”.

### 2.3 Data analysis

Participants were grouped according to current or previous treatment seeking behaviour. Thus, any participant who answered yes to either question was allocated to the ‘current / previous treatment seeking’ group. Participants who answered no to both questions were allocated to the ‘no treatment seeking’ group.

A repeated-measures ANOVA with time (4 timepoints) as the within-subjects factor and treatment seeking (yes/no) group (2 groups) as the between-subjects factor was first conducted. This was followed by the same analysis on the change-from-T1 scores. These change scores were calculated by subtracting the baseline score from the score at the subsequent timepoint (2, 3 and 4) (i.e., T2-T1, T3-T1, T4-T1). This repeated-measures ANOVA with the mean change scores therefore had 3 timepoints (T2, T3, T4) for the within-subjects factor. This approach has been deemed to be more reliable when controlling for baseline differences in non-randomised studies with pre-existing groups [29]. An a priori power analysis was conducted using G*Power to determine the minimum sample size required. For a medium effect size, at power of 0.8 and alpha of 0.05, our analysis suggested a minimum requirement of 28 participants per group.

## 3 Results

### 3.1 Participants

Participants were 167 cisgender females, between 18-65 years of age (mean = 36.38, SD = 14.22), who completed all four timepoints of data collection. The majority of the participants were White (92.2%; n=153). At T1, 65.9% were employed or self-employed (n=110) and 25.8% were in education (n=43). Only one person was unemployed and looking for work, while three (1.8%) classified themselves as a homemaker and 10 (n=6%) were retired, not looking for work, or ‘other’. For the majority of participants (n=150, 89.8%), their employment status was the same as pre-pandemic. Most participants were living with a partner; either married or in a civil partnership (n=68, 40.7%) or co-habiting (n=30, 18%). Twenty-one (12.6%) were in a non-co-habiting relationship, while 44 (26.3%) were single, four (1.8%) were widowed and one ‘other’. Approximately one-third (n=46, 27.6%) of the participants had at least one child living with them.

Based on responses to the question regarding treatment-seeking behaviour for a mental health issue, 59 participants were grouped into the “current / previous treatment” group (mean age = 34.49) and 108 participants were grouped into the “no treatment” group (mean age = 37.42; *t*[1,165]= −1.27, *p* = 0.205)

### 3.2 Wellbeing over 12 months

The repeated-measures ANOVA on the mean scores on each mental wellbeing measure at each timepoint (T1-T4) found that there was only one main effect of Time and this was on symptoms of depression. However, there was a main effect of Treatment Seeking (yes/no) on all three measures; anxiety, depression, and trauma. As expected, those who reported current / previous treatment-seeking scored significantly higher than those in the no treatment seeking group, indicating poorer wellbeing. See Table 1 for mean (SD) mental health scores at each timepoint by treatment group.

**Table 1:** Mean (SD) wellbeing scores at each timepoint and ANOVA statistic.

	Current/Previous Treatment Seeking (n= 59)				No Treatment Seeking (n= 108)				*F*	
	T1	T2	T3	T4	T1	T2	T3	T4	Group	Time
HADS - Anxiety	9.14 (4.60)	9.00 (4.55)	9.05 (6.86)	8.73 (4.58)	6.83 (3.53)	6.35 (4.15)	6.86 (4.17)	6.73 (4.29)	14.883⁎⁎⁎	0.565
HADS - Depression	7.08 (3.85)	6.68 (4.24)	6.29 (4.72)	7.37 (4.75)	4.81 (3.35)	4.43 (3.49)	4.56 (3.68)	5.71 (3.83)	14.159⁎⁎⁎	6.256⁎⁎⁎
SQD - Trauma	4.27 (2.23)	3.93 (2.39)	4.12 (2.43)	4.14 (2.40)	3.18 (2.10)	3.08 (2.35)	3.09 (2.49)	3.26 (2.37)	8.438⁎⁎⁎	0.750

⁎⁎⁎ p < 0.001

### 3.3 Change in wellbeing

**Fig. 1:**
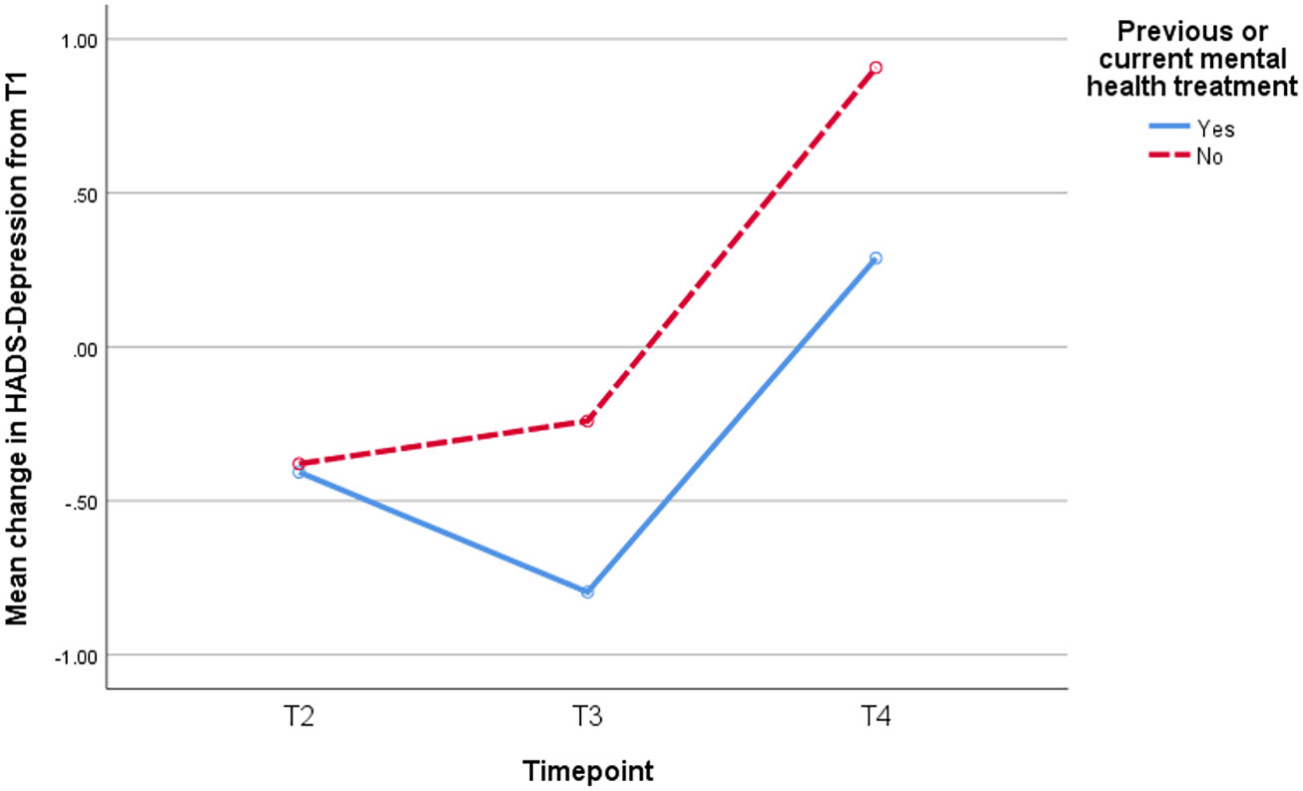
Mean change in depression from T1 (baseline) at T2, T3, and T4 in participants who are currently or in the past sought treatment for their mental wellbeing and those who have never sought treatment.

In order to control for the baseline differences in anxiety, depression, or trauma symptoms between treatment seeking groups, and to compare the magnitude of change experienced within each group, a repeated-measures ANOVA was conducted on the change scores (T2/T3/T4 – T1). No differences between groups were found on any measure (see Table 2). However, a significant main effect of Time was found for the changes in depression (*F*[57.09, 2] = 9.314, *p* < 0.001). See Fig. 1. No other significant main effects or interactions were observed.

**Table 2:** Mean (SD) change in wellbeing scores from baseline at timepoint 2, 3 and 4 and ANOVA statistic.

	Current / Previous Treatment Seeking (n= 59)			No Treatment Seeking (n= 108)			*F*	
	T2-T1	T3-T1	T4-T1	T2-T1	T3-T1	T4-T1	Group	Time
HADS - Anxiety	−.136 (3.79)	−.085 (3.83)	−.407 (4.23)	−.482 (3.19)	.028 (3.23)	−.102 (3.63)	0.003	0.028
HADS - Depression	−.407 (3.25)	−.797 (3.74)	.288 (4.30)	−.380 (2.68)	-.241 (3.70)	.907 (3.60)	0.742	9.314⁎⁎⁎
SQD - Trauma	−.339 (2.19)	−.153 (2.38)	−.136 (2.33)	−.037 (1.77)	−.028 (1.98)	.139 (2.08)	0.654	0.816

⁎⁎⁎ p < 0.001

## 4 Discussion

This study is the first to track changes in wellbeing between previous / current mental health treatment seekers and non-treatment seekers, longitudinally over the first year of the COVID-19 pandemic. Unsurprisingly, those who had previously / were currently seeking treatment for mental health issues reported significantly greater symptoms of anxiety, depression, and disaster-related trauma reactions at all timepoints than those who had not sought any treatment, which is consistent with other research (e.g., [30]). In addition, for both groups, depression changed significantly over time; initially decreasing from the start of the pandemic and increasing to highest levels at the final timepoint, following a third national lockdown. However, this study extends previous work via analysis of change scores which revealed no significant difference between groups in the magnitude of change experienced on any wellbeing measure, including depression. This suggests that while the high-risk (i.e. treatment seeking) group were, and remained, higher in depression, the trajectory of their experience mirrored that of the low-risk group. This is significant as it suggests that all females in this study experienced a similar shift in their wellbeing, irrespective of risk status. This can be interpreted in two ways. Positively, it suggests that high-risk individuals did not experience greater negative effects (in terms of magnitude of wellbeing changes) than low-risk individuals, perhaps illustrating the effectiveness of current strategies to manage their wellbeing. However, conversely, it may suggest that low-risk individuals experienced changes of the same magnitude as those who are more vulnerable, and if so, this is of great concern. Indeed, the data show a trend for those with no previous treatment-seeking behaviour to experience a greater increase in depression over time. This may be because they are less well-equipped with protective psychological strategies to deal with the significant negative impacts of COVID-19 [31].

Indeed, a recent study in Germany suggested increased alcohol consumption among women, particularly those who were mothers, to be a sign of “self-treatment” to cope with the stressors and burden of the pandemic, while experience of past stressful events was deemed to provide some protection by learning from experience [32]. Indeed, a recent study reported that maladaptive avoidance was associated with anxiety and depression during the pandemic [33] and another reported that the likelihood of developing acute stress disorder during the COVID-19 pandemic was only mediated by the presence of adaptive coping strategies (and not the absence of maladaptive ones; [34]). This suggests that the benefit of having very good coping strategies may be greater than the drawback of having maladaptive ones or none at all. Here, we are suggesting that previous / current treatment seeking behaviour may serve as a protective factor, by facilitating the development of adaptive coping strategies via contact with a mental health professional or GP, and by having an increased awareness of changes in, and the management of, their wellbeing. Such a conclusion suggests that knowledge and practice in adaptive mental health support strategies are effective preventative and protective measures and that education surrounding this should perhaps be regarded as a fundamental life skill, similar to bodily First Aid training.

The Mental Health First Aid (MHFA) programme has been shown to be effective in increasing knowledge of mental health problems, reducing stigma, and increasing support given to those experiencing problems [35,36] and can thus be regarded as a valuable public health intervention. Indeed, these findings suggest that had our no treatment group been equipped with strategies to deal with changes in their mental health they may not have experienced similar changes in wellbeing as our treatment-seeking group. However, a limitation of the study and our categorisation based on treatment-seeking behaviour is that we do not know for certain whether our no treatment-seeking group have not previously sought treatment because they have never experienced low wellbeing, or because they lack the insight or resource to be able to do so. Furthermore, we do not know exactly what treatment the treatment-seeking group may have received. However, while we do not have information on each individual’s treatment-seeking motivators, experiences, severity of any current / previous problems, and mental health knowledge, this hypothesis remains worthy of consideration. Indeed, it suggests that if all individuals were educated and proficient in adaptive coping and mental health strategies, the need for treatment over the long term may be reduced. Since mental health costs have recently been estimated, conservatively, to cost the UK National Health Service £125 billion per year, this preventative approach could prove to be extremely financially beneficial [37].

This study only explored the experiences of females across the first year of the pandemic. This is because it is well documented that many of the additional and intersecting burdens of the pandemic fell disproportionately on women, often exacerbating existing structural inequalities (e.g., in childcare, healthcare, government representation; [38]), and that men and women reacted to, and coped with, the pandemic in different ways [39]. Therefore, while we cannot draw any conclusions regarding the generalisability of this finding to other cohorts within the population, our focus is founded in the need to understand women’s experiences specifically and unapologetically. That said, a limitation of the study is the lack of ethic and socioeconomic diversity within our sample of women, being mainly White, employed, married/cohabiting, and with no children living in the home, which limits the generalisability of the findings to all women. While a strength of this study is the simplicity of the approach to compare the effect of current/previous treatment versus no treatment on wellbeing across time, a deeper investigation into additional variables that may be influencing the change in wellbeing reported over the 12-month period (e.g., employment status, family dynamics, health and wellbeing behaviours etc) would be of interest. Indeed, that we do not have more information on demographics that may affect vulnerability between women, for example educational background or financial instability, is an acknowledged limitation of this study. Despite this, the findings reported here provide a compelling conclusion that experiences of poor mental wellbeing and treatment-seeking behaviour do not pose an inevitable vulnerability to further negative impact on wellbeing in the context of significant and unprecedented life events. Rather, those with no previous treatment-seeking behaviour may be, ironically, more vulnerable.

Acknowledging and understanding these differentiating impacts is critical for re-thinking the importance of preventative approaches to mental health problems and ensuring adequate preparation to give appropriate support to individuals moving forward, both from the pandemic and more generally. Specifically, a lot can be gained from research that is guided by, and draws upon, lived experiences [40]. That is, the first-hand understanding of how mental health is impacted and protected/restored by those who have been, and continue to be, affected. Importantly, this often includes insight into the barriers to efficacy of treatments and the influence of those delivering them e.g., mental health nurses [41]. Indeed, lived-experiences are considered important in helping to shape social policy [42] and have an important role to play in developing interventions to support COVID recovery, particularly. Without experience-led, intersectionally-informed insight, interventions are unlikely to be wholly effective due to lack of specificity and applicability. Notwithstanding this, future research that also focuses on exploring how (and why) different populations develop and hone coping skills and identifying how these behaviours can be modelled / taught to others would be an important step in supporting wider population wellbeing.

Overall, these findings should be taken as a call to action to extend more support to the general population, who may be experiencing relatively greater distress by virtue of their previous lack of need and contact with support services. Indeed, with the rising levels of mental health problems in society generally, there is a need to equip people with the skills to cope.

## Funding

No funds, grants, or other support was received to conduct this study or prepare the manuscript.

## Declaration of Competing Interest

The authors have no relevant financial or non-financial interests to disclose.
